# Short-term high-fat diet feeding plus acute ethanol binge induced acute liver injury in mice via oxidative stress, inflammation and pyroptosis

**DOI:** 10.3389/fphar.2025.1602280

**Published:** 2025-06-18

**Authors:** Yao Deng, Xinling Chen, Wenhai Guo, Yun Chen, Luyao Xu, Wenting Suo, Wei Liu, Jiaying Dai, Kangrong Wang, Qiuling Li, Chengqin Lu, Min Dai, Jiean Xu, Jinwen Xu, Hequan Zhu, Zaoyuan Kuang, Yaxing Zhang

**Affiliations:** ^1^ Research Centre of Basic Integrative Medicine, School of Basic Medical Sciences, Guangzhou University of Chinese Medicine, Guangzhou, China; ^2^ Department of Physiology, School of Basic Medical Sciences, Guangzhou University of Chinese Medicine, Guangzhou, China; ^3^ Department of Traditional Chinese Medicine, The Third Affiliated Hospital, Sun Yat-sen University, Guangzhou, China; ^4^ Department of Gynecology, The Second Clinical School of Guangzhou University of Chinese Medicine, The Second Affiliated Hospital of Guangzhou University of Chinese Medicine, Guangdong Provincial Hospital of Chinese Medicine, Guangzhou University of Chinese Medicine, Guangzhou, China; ^5^ Institute of Integrated Traditional Chinese and Western Medicine, Sun Yat-sen University, Guangzhou, China; ^6^ Department of Allergy and Immunology, The Third Affiliated Hospital, Sun Yat-sen University, Guangzhou, China; ^7^ Luzhou Key Laboratory of Research for Integrative on Pain and Perioperative Organ Protection, Department of Anesthesiology and Pain, The Affiliated Traditional Chinese Medicine Hospital, Southwest Medical University, Luzhou, China

**Keywords:** high-fat diet feeding, acute ethanol binge, oxidative stress, caspase-1, caspase-8, caspase-11, GSDMD, GSDME

## Abstract

**Background:**

Ethanol binge and obesity are the key risk factors for alcohol-related liver disease (ALD) and nonalcoholic fatty liver disease (NAFLD), respectively. The human beings have a habit of drinking alcohol and consuming high calorie foods, these two factors often coexist, and thus contributing to the liver injury. However, the mechanisms of a short-term consumption of high-fat diet (HFD) plus alcohol binge-induced acute liver injury are unclear.

**Methods:**

Male C57BL/6 mice (aged 8–10 weeks) were fed a HFD or HFD Control diet for 3 days. Then, they received a single dose of ethanol or the same volume of distilled water by oral gavage. The liver damage was evaluated after 9 h of ethanol gavage.

**Results:**

Short-term (3 days) HFD feeding plus ethanol binge significantly aggravated liver injury and steatosis in mice as indicated by the increased serum alanine aminotransferase (ALT), aspartate aminotransferase (AST), and triglyceride (TG) levels, the upregulated hepatic TG levels, and Oil Red O staining and H&E staining. Mechanistically, short-term HFD feeding plus ethanol binge disturbed hepatic redox homeostasis by increasing 3-nitrotyrosine (3-NT), malondialdehyde (MDA) and myeloperoxidase (MPO) levels, while decreasing glutathione (GSH) levels. HFD and alcohol co-consumption also increased hepatic TNF-α, IL-1β and IL-18 via enhancing the phosphorylation of MAPK (ERK1/2, p38 and JNK) and NF-κB. The canonical (Caspase-1 to GSDMD) and non-canonical pyroptosis signaling (Caspase-8/11 to GSDMD, and Caspase-3 to GSDME) further contributed to the acute liver injury.

**Conclusion:**

Short-term HFD feeding plus a single dose of ethanol gavage can significantly exacerbate acute liver injury and hepatic fat deposition in mice by enhancing oxidative stress, MAPK and NF-κB signaling, and Caspase-1/8/11-GSDMD and Caspase-3-GSDME pyroptosis signaling.

## 1 Introduction

Alcohol-related liver disease (ALD) and nonalcoholic fatty liver disease (NAFLD, currently known as metabolic dysfunction-associated steatotic liver disease, MASLD) are the leading causes of chronic liver disease worldwide (Díaz et al., 2023; Matchett et al., 2024). ALD and NAFLD share pathophysiological, histological, and genetic features and both alcohol and metabolic dysfunction coexist as aetiological factors in many patients with hepatic steatosis (Chao et al., 2023; Díaz et al., 2023). Currently, approximately 2 billion people consume alcohol worldwide and upwards of 75 million are diagnosed with alcohol-use disorders and are at risk of ALD, moreover, about 2 billion adults are obese or overweight and over 400 million have diabetes, both of which are the risk factors for NAFLD and hepatocellular carcinoma (Asrani et al., 2019). Although diagnosis of NAFLD requires the exclusion of significant alcohol consumption and other causes of liver disease, significant alcohol consumption is often under-reported in NAFLD patients and that metabolic factors and alcohol interact to exacerbate the progression of liver disease (Díaz et al., 2023).

In modern society, people often drink alcohol to relieve stress and obtain entertainment. At the same time, alcohol culture is also a major means of social interaction. Importantly, binge drinking and high-calorie eating often coexist, these two factors synergistically cause and aggravate the liver damage. Short-term high-fat diet (HFD) feeding plus acute ethanol binge have a synergistic effect on the liver injury (Chang et al., 2015). However, the potential molecular mechanisms of acute liver injury caused by high-calorie eating and alcohol binge consumption still need to be further explored. Here, we used an acute liver injury model in mice fed with HFD for 3 days plus a single binge of ethanol to investigate the synergistic mechanism of HFD plus alcohol binge on acute liver injury.

## 2 Methods

### 2.1 Animal models

The male C57BL/6 mice were purchased from Guangdong Medical Laboratory Animal Center (Foshan, China), they were housed in a temperature-controlled animal facility with a 12-h light–dark cycle and allowed to obtain rodent chow and water *ad libitum*. All experimental procedures of animals in this study were approved by the Institutional Animal Care and Use Committee of Guangzhou University of Chinese Medicine.

**FIGURE 1:**
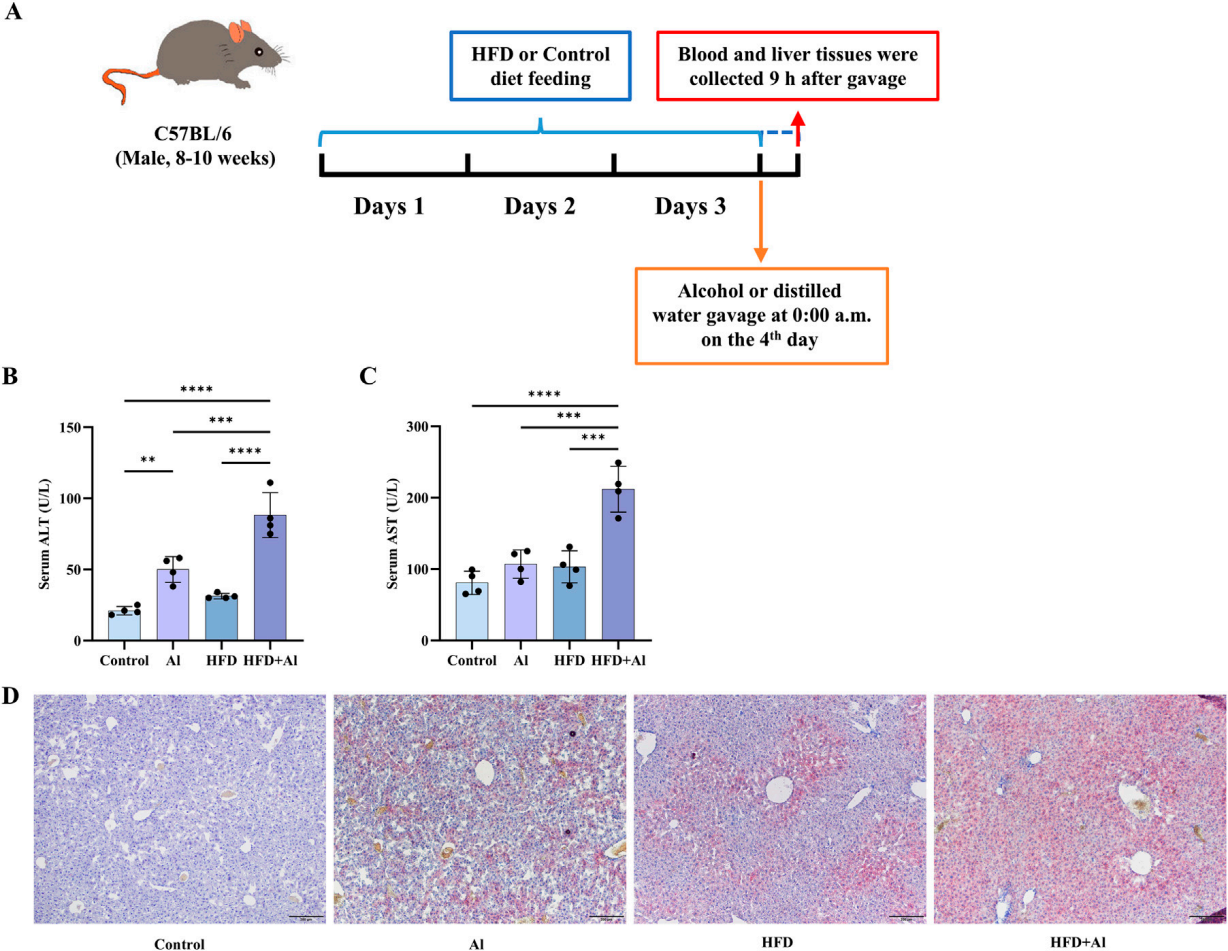
HFD feeding for 3 days plus one acute ethanol binge synergistically induced acute liver injury in mice. **(A)** Male C57BL/6 wild-type mice started feeding a HFD diet or a Control diet at 9:00 p.m. before the first day, and lasted for 3 days. Then, a single dose of ethanol (5 g/kg body weight as a 31.25% ethanol in water; 0.02 mL/g) was given to mice via gavage at 0:00 a.m. on day 4. The food was not taken away after gavage. The liver and blood were collected at 9:00 a.m. on the fourth day. **(B)** Serum ALT levels. **(C)** Serum AST levels. **(D)** Hepatic Oil Red O staining. All data were expressed as mean ± SD, n = 4 in each group, ^**^p < 0.01, ^***^p < 0.001, ^****^p < 0.0001.

The animal model was established according to Gao’s group previously described with appropriate modifications (Chang et al., 2015; Ding et al., 2010). In detail, the animals were fed a HFD (60% kcal% fat; Cat# GD60, Guangdong Medical Laboratory Animal Center, Foshan, China; Table 1) or a HFD Control diet (10% kcal% fat; Cat# GD450B, Guangdong Medical Laboratory Animal Center, Foshan, China; Table 1) for 3 days, followed by a single gavage of ethanol (as a 31.25% solution in water) at a dose of 5 g/kg body weight (as 0.20 mL per 10 g body weight) or the same volume of distilled water (Chang et al., 2015). The food was not taken away after gavage. After 9 h of ethanol gavage, the animals were deeply anesthetized before blood collection from the orbital sinus for collecting serum and euthanized via cervical dislocation (Mackowiak et al., 2022) (Figure 1A). Then, the liver samples were harvested, and either frozen in −80°C or put in 4% Paraformaldehyde Fix Solution (Cat# G1101, Servicebio, Wuhan, China) before further analysis.

**TABLE 1:** The diet formula.

Ingredient	HFD Control		HFD	
	Weight ratio %	Energy ratio %	Weight ratio %	Energy ratio %
Protein	19.2	20	26	20
Carbohydrate	67.3	70	26	20
Fat	4.3	10	35	60
Total		100		100

### 2.2 Biochemical analysis

Serum levels of alanine aminotransferase (ALT), aspartate aminotransferase (AST), and triglycerides (TG) were examined by an automatic biochemical analyzer as we had previously descried (Xu et al., 2024). Hepatic TG, malondialdehyde (MDA), myeloperoxidase (MPO) and the reduced glutathione (GSH) were examined by the commercial assay kits (Cat# A110-1-1, Cat# A003-1-2, Cat# A044-1-1, and Cat# A006-2-1, Nanjing Jiancheng Bioengineering Institute, Nanjing, China) (Xu et al., 2024).

### 2.3 Histopathology analysis

The fixed liver samples were embedded by the Tissue-Tek^®^ optimum cutting temperature (O.C.T.) compound (Cat# 4583, Sakura, Japan) to perform frozen liver sections for Oil Red O (Cat# G1015, Servicebio, Wuhan, China) staining, or embedded in paraffin to perform sections for staining with hematoxylin (Cat# Ba4097, BaSo, Zhuhai, China) and eosin (Cat# Ba4099, BaSo, Zhuhai, China) (H&E) as we previously described (Zhang et al., 2020; Xu et al., 2024). H&E staining was used to evaluate the morphological change of the liver tissue and lipid accumulation, and Oil Red O staining was used to visualize fat content in the liver samples.

### 2.4 Immunoblot assay

The total proteins were extracted from the liver samples with lysis buffer, and immunoblot assay was performed according to the standardized processes (Zhang et al., 2020). The antibodies used in this study were described in Table 2. The gray value of protein bands was quantified by the ImageJ software (National Institutes of Health, Bethesda, MD, United States).

**TABLE 2:** The antibodies information.

The antibody	The product number	The manufactures
Phospho- NF-κB p65 antibody	#3033S	Cell Signaling Technology (Danvers, MA, United States)
NF-κB p65 antibody	#8242S	
JNK antibody	#9252S	
p38 MAPK antibody	#8690S	
Phospho-p38 MAPK antibody	#4511S	
Erk1/2 antibody	#4695S	
phospho-JNK antibody	#9255S	
Caspase-8 antibody	#9746	
TNF-α antibody	#AF7014	Affinity Biosciences (Changzhou, Jiangsu, China)
Pro-IL1-β Antibody	#AF5103	
Cleaved-IL1-β Antibody	#AF4006	
Goat Anti-Rabbit IgG (H + L) HRP	#S0001	
Goat Anti-Mouse IgG (H + L) HRP	#S0002	
Caspase-3 antibody	#sc-56053	Santa Cruz Biotechnology (Santa Cruz, CA, United States)
Caspase-11 antibody	#sc-374615	
Caspase-1 antibody	#ab1872	Abcam (Cambridge, United Kingdom)
GSDMD antibody	#ab219800	
GSDME antibody	#ab215191	
GAPDH antibody	#MB001	Bioworld, Technology (Qixia District, Nanjing, China)
IL-18 antibody	#D046-3	Medical & Biological Laboratories Co., Ltd (Tokyo, Japan)
p-ERK1/2 antibody	#bs-3016R	Bioss Antibodies (Beijing, China)

### 2.5 Immunofluorescence staining

Immunofluorescence staining was performed to detect 3-nitrotyrosine (3-NT) in the liver. The frozen sections were subjected to pre-washing treatments as follows: washing with 1 × PBS for 30 min, 1 × TBS for 10 min, and 1 × TBST for 20 min. The liver sections were sealed with goat serum blocking solution (Cat# ZLI-9056, ZSGB-BIO, Beijing, China) at room temperature for 2 h. Then, the 3-NT antibody (Abcam, Cat# ab110282, United Kingdom) was incubated overnight with the liver sections at 4°C, followed by washing with 1 × TBS for 10 min and 1 × TBST for 20 min. The liver sections were incubated with Goat Anti-Mouse IgG (H + L) Fluor 594-conjugated antibody (Cat# S0005, Affinity Biosciences, Beijing, China) under dark conditions for 2 h. The sections were washed with 1 × TBS for 10 min followed by 1 × TBST for 20 min. Finally, the fluorescence intensity of 3-NT was observed under a fluorescence microscope after sealing the sections with anti-fluorescence attenuation sealer (containing DAPI) (Cat# S2110, Solarbio, Beijing, China).

### 2.6 Statistical analysis

The statistical analyses were performed by one-way analysis of variance (ANOVA) followed by Bonferroni’s *post hoc* analysis for data with normal distribution (by Shapiro-Wilk test) and satisfying homogeneity of variance (by Brown-Forsythe test), performed by Brown-Forsythe and Welch ANOVA tests followed by Dunnett T 3 *post hoc* analysis for data with normal distribution and heteroscedasticity, and performed by Kruskal–Wallis test followed by Dunn’s *post hoc* analysis for data with skewed distributions (by Shapiro-Wilk test). All data were expressed as the mean ± SD or median ± interquartile range, a value of P < 0.05 was considered as significantly different. All histograms were performed using GraphPad Prism 10.0 (GraphPad Software Inc., San Diego, CA, United States).

## 3 Results

### 3.1 Short-term HFD feeding plus acute ethanol binge induced acute liver injury in mice

It has been reported that 3 days of HFD feeding plus acute ethanol binge can induce liver damage in mice (Chang et al., 2015; Babuta et al., 2024c). Here, we established the same animal models (Figure 1A), and like the previous reports (Chang et al., 2015; Babuta et al., 2024c), our data also indicated that HFD feeding for 3 days plus one time of acute ethanol binge on the third day synergistically induced liver injury as indicated by increasing serum levels of ALT and AST (Figures 1B,C) and increasing hepatic fat deposition in mice as indicated by Oil Red O staining (Figure 1D).

**FIGURE 2:**
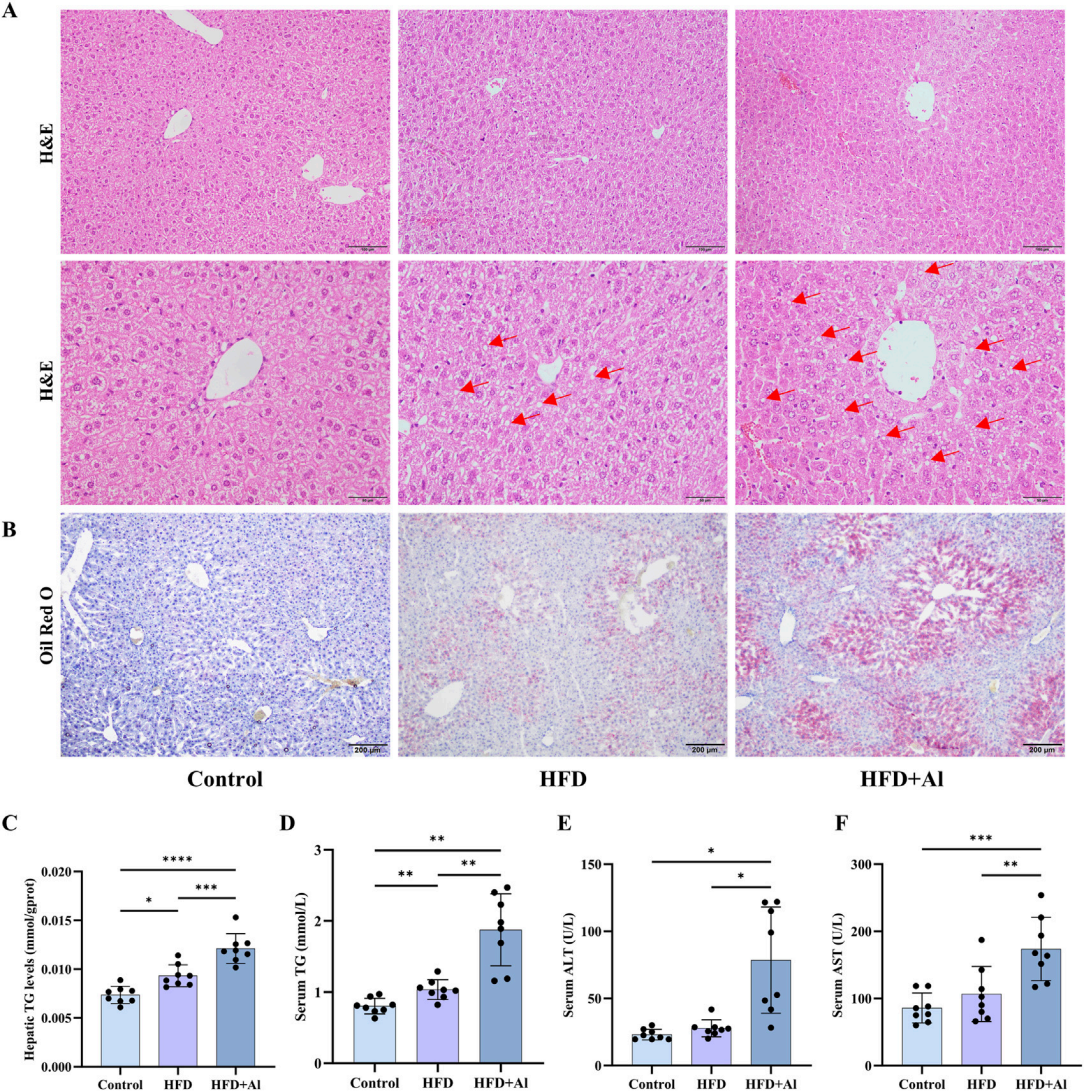
Short-term HFD feeding plus acute ethanol gavage induced acute hepatic steatosis in mice. **(A)** The H&E staining of the liver sample. **(B)** The Oil Red O staining of the liver sample. **(C)** Hepatic TG levels in mice. **(D)** The serum TG levels. **(E)** The serum ALT levels. **(F)** The serum AST levels. All data were expressed as mean ± SD, n = 8 in each group, ^*^p < 0.05, ^**^p < 0.01, ^***^p < 0.001, ^****^p < 0.0001.

In order to further investigate the mechanisms of short-term HFD feeding plus acute ethanol binge on acute liver injury, the other animals were randomly divided into three groups: Control group, HFD group, and short-term HFD feeding plus acute ethanol binge group. Similar to the previous reports that short-term HFD feeding, e.g., for 3–4 days, is sufficient to induce liver steatosis and impair glucose tolerance and hepatic insulin sensitivity (Lee et al., 2011; Ji et al., 2012; Wiedemann et al., 2013; Chang et al., 2015), our data of hepatic H&E staining showed that a small amount of fat vacuoles accumulated in the liver of mice fed a HFD (Figure 2A), hepatic Oil red O staining and hepatic TG levels showed that HFD feeding induced fat deposition in the liver (Figures 2B,C). HFD feeding also increased serum TG levels, and only slightly increase serum ALT and AST levels with no statistical significance (Figures 2D–F). Compared with HFD group, hepatic fat deposition, serum TG, ALT and AST levels were further enhanced in HFD + Al group (Figure 2). Therefore, short term HFD feeding plus ethanol binge synergistically induces acute liver injury and hepatic steatosis in mice.

### 3.2 HFD plus ethanol binge synergistically induced oxidative stress in the liver

**FIGURE 3:**
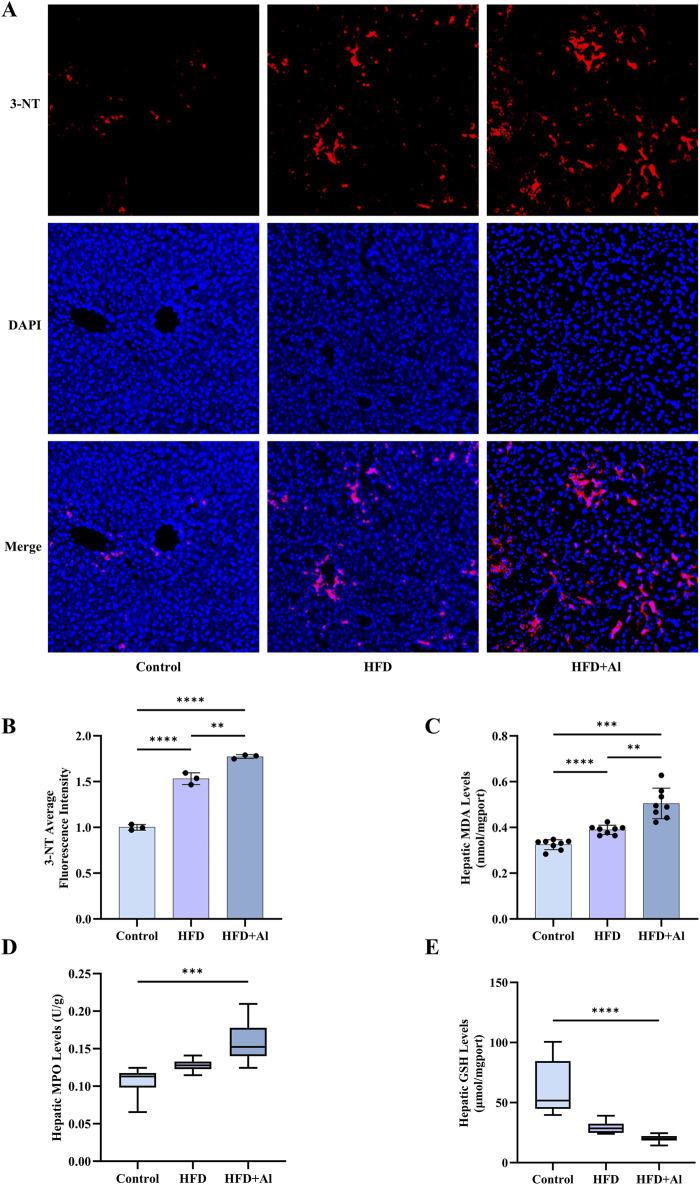
Short-term HFD feeding plus acute ethanol binge induced hepatic oxidative stress in mice. **(A)** The immunofluorescence staining of 3-NT, 10 ×. **(B)** The average fluorescence intensity of 3-NT, n = 3 in each group. **(C)** Hepatic MDA levels; **(D)** Hepatic MPO levels; **(E)** Hepatic GSH levels. The data of MPO and GSH were expressed as median ± interquartile range, and the other data were expressed as mean ± SD, n = 8 in each group in C to E, ^**^p < 0.01, ^***^p < 0.001, ^****^p < 0.0001.

HFD or excessive ethanol intake has been shown to promote the production of reactive oxygen species (ROS), thus, increase oxidative stress in the liver (Ma et al., 2022; Zhu et al., 2022; Park et al., 2023). The high levels of ROS can induce lipid peroxidation. 3-nitrotyrosine (3-NT), a product of reactive-nitrogen species (RNS) with the activated aromatic ring of tyrosine, is another classical biomarker of oxidative stress (Bandookwala and Sengupta, 2020). The immunofluorescence results indicated that hepatic 3-NT levels in HFD group and HFD + Al group were increased when compared with Control group, and hepatic 3-NT levels in HFD + Al group were higher than those in HFD group (Figures 3A,B). MDA, which is a biomarker of lipid peroxidation, has been examined to reflect oxidative stress (Ayala et al., 2014). HFD, and HFD plus ethanol binge increased MDA levels in the liver, and HFD plus ethanol binge upregulated more MDA levels in the liver (Figure 3C). MPO, which is the member of heme peroxidase family in immune cells, can contribute to ROS production (Hawkins and Davies, 2021), and thus, MPO levels also reflects the degree of oxidative stress and inflammation in the organ or tissue. Our data indicated that hepatic MPO levels were increased (Figure 3D), in contrast, GSH (an indicator of antioxidant capacity) levels were decreased in HFD + Al group compared with Control group (Figure 3E). Therefore, HFD plus ethanol binge synergistically contributed to hepatic oxidative stress in mice.

### 3.3 HFD plus ethanol binge upregulated MAPK and NF-κB phosphorylation in the liver

**FIGURE 4:**
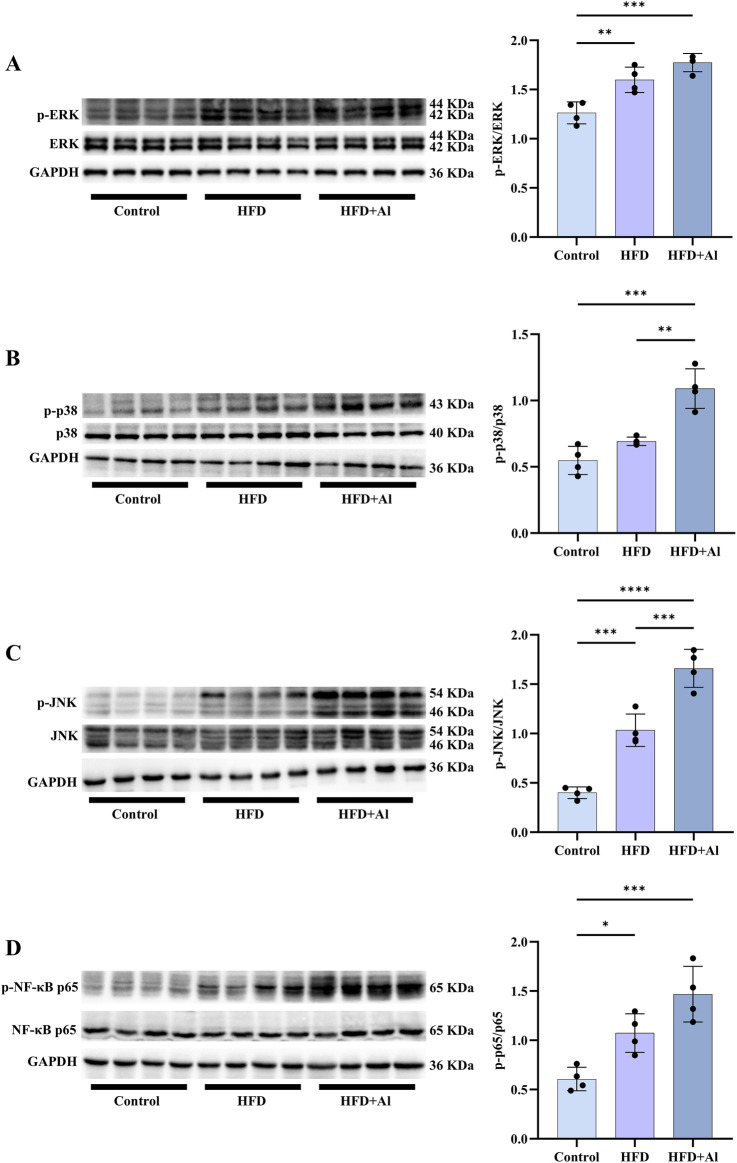
Short-term HFD feeding plus acute ethanol binge enhanced the phosphorylation of MAPK and NF-κB p65 in the liver of mice. Western blotting and quantification of p-ERK/total ERK **(A)**, p-p38/total p38 **(B)**, p-JNK/total JNK **(C)**, and p-NF-κB p65/total NF-κB p65 **(D)**. All data were expressed as mean ± SD, n = 4 in each group, ^*^p < 0.05, ^**^p < 0.01, ^***^p < 0.001, ^****^p < 0.0001.

The increased ROS can further cause acute liver injury after short-term ethanol feeding by activating innate immune signals and inducing sterile inflammation (Tsutsumi et al., 2003; Gao et al., 2024). Here, the phosphorylation of MAPK and NF-κB p65 in the liver were evaluated by Western blotting. Our results showed that the phosphorylation of hepatic ERK, JNK, and NF-κB p65 were elevated in HFD group when compared with Control group, and the phosphorylated hepatic MAPK (p38 and JNK) were further increased in the mice subjected to HFD plus ethanol binge, and ERK and NF-κB p65 also showed increasing trends, however, it was not statistically significant (Figure 4). Therefore, HFD plus ethanol binge significantly increased the phosphorylation levels of MAPK and NF-κB in the liver.

### 3.4 HFD plus ethanol binge aggravated inflammatory cytokines expression in the liver

**FIGURE 5:**
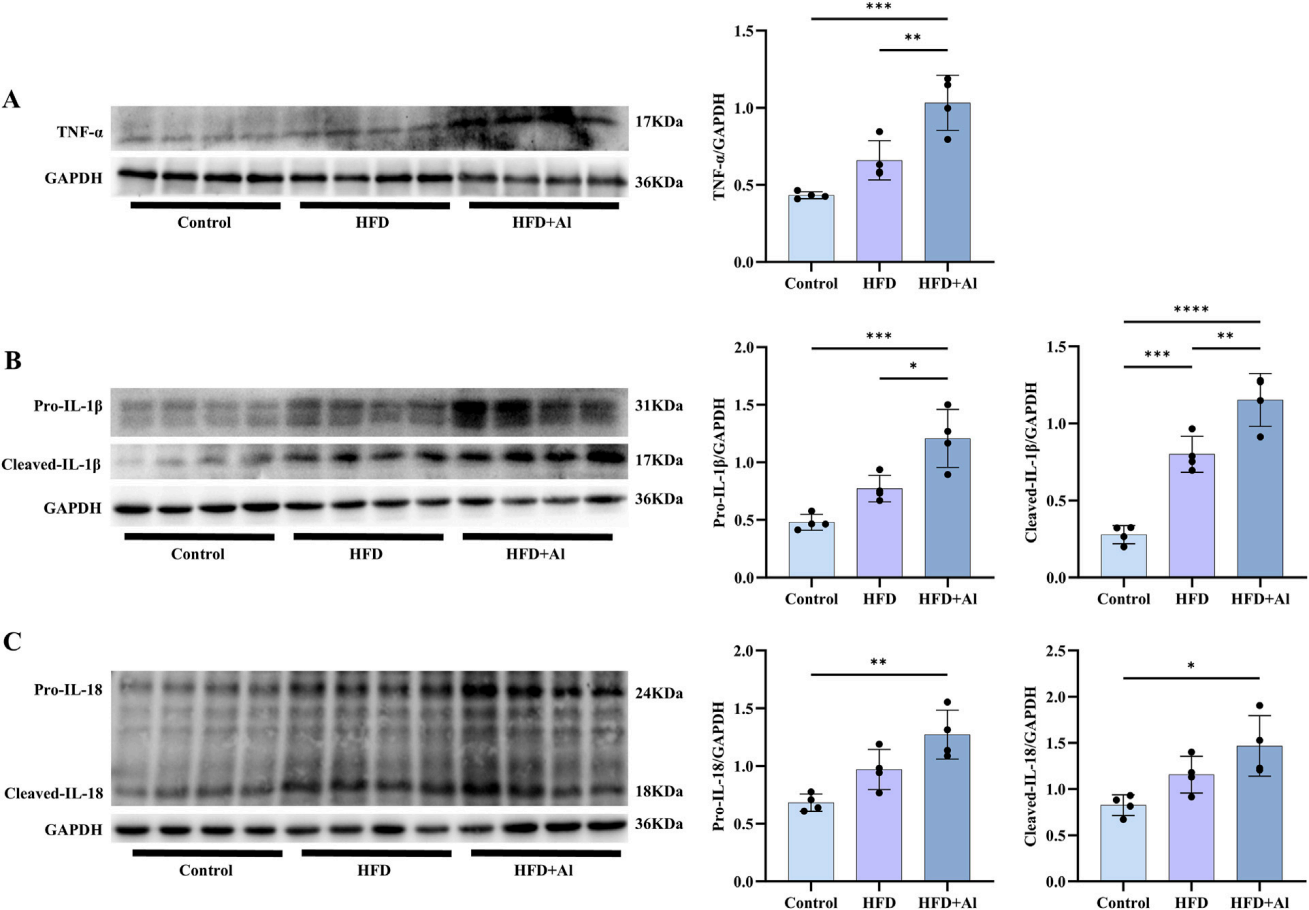
Short-term HFD plus acute ethanol binge enhanced the expression and maturation of hepatic TNF-α, IL-1β and IL-18 in mice. Western blotting and quantification of TNF-α/GAPDH **(A)**, pro-IL-1β/GAPDH and cleaved-IL-1β/GAPDH **(B)**, and pro-IL-18/GAPDH and cleaved-IL-18/GAPDH **(C)**. All data were expressed as mean ± SD, n = 4 in each group, ^*^p < 0.05, ^**^p < 0.01, ^***^p < 0.001, ^****^p < 0.0001.

The phosphorylation of MAPK and NF-κB p65 in the liver can induce the expression of inflammatory cytokines, and thus amplifying inflammation (Zhang et al., 2019; Zhang et al., 2023; Lan et al., 2024). Therefore, TNF-α, IL-1β, and IL-18 levels in the liver were determined by Western blotting. Our results showed that the hepatic protein levels of TNF-α, pro-IL-1β and the cleaved-IL-1β (the maturation form of IL-1β), and pro-IL-18 and the cleaved-IL-18 (the maturation form of IL-18) were increased in HFD + Al group compared with HFD group (IL-18 levels only show upward trends but has no statistical significance) (Figure 5). Therefore, HFD plus ethanol binge induced and amplified hepatic inflammation by aggravating both the expression and maturation of inflammatory cytokines in mice.

### 3.5 HFD plus ethanol binge synergistically induced hepatic pyroptosis

**FIGURE 6:**
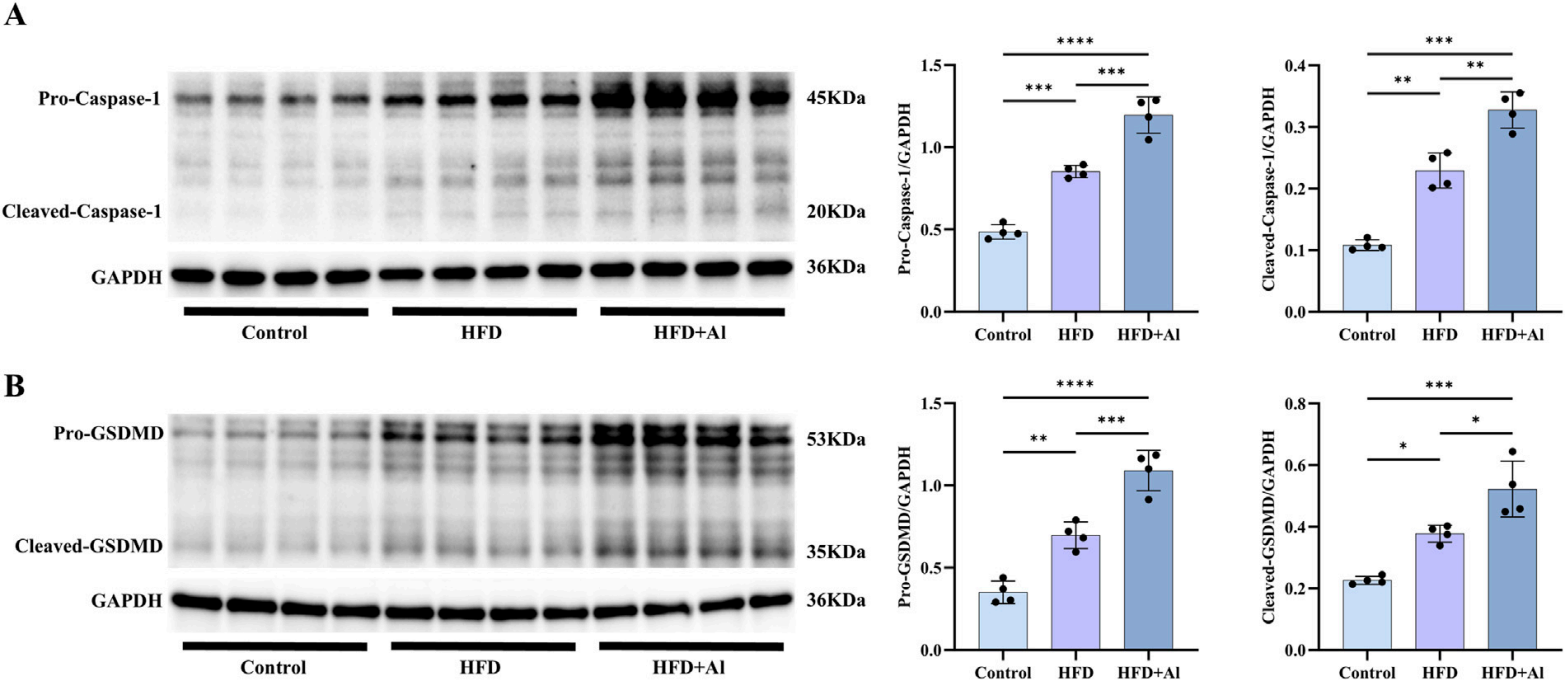
Short-term HFD feeding plus acute ethanol binge activated the canonical Caspase-1 to GSDMD pyroptosis signaling in the liver of mice. Western blotting and quantification of pro-Caspase-1/GAPDH and cleaved-Caspase-1/GAPDH **(A)**, and pro-GSDMD/GAPDH and cleaved-GSDMD/GAPDH **(B)**. All data were expressed as mean ± SD, n = 4 in each group, ^*^p < 0.05, ^**^p < 0.01, ^***^p < 0.001, ^****^p < 0.0001.

The pro-IL-1β and pro-IL-18 need to be processed and cleaved by the activated Caspase-1 (the cleaved Caspase-1) to convert into their mature forms (He et al., 2016). Therefore, to investigate whether the effect of HFD plus ethanol binge on the maturation of IL-1β and IL-18 was related to the activation of Caspase-1, we examined the protein levels of Caspase-1. Our results showed that both pro-Caspase-1 and cleaved-Caspase-1 were upregulated in HFD group when compared with Control group, and HFD plus ethanol binge further increased the expression and the activation of Caspase-1 (Figure 6A). Besides the cleavage of IL-1β and IL-18, Caspase-1 can also specifically cleave the linker between the amino-terminal gasdermin-N and carboxy-terminal gasdermin-C domains in gasdermin D (GSDMD), which is required and sufficient for pyroptosis (Shi et al., 2015). To further investigate whether HFD plus ethanol binge-induced acute liver injury is involved in pyroptosis, we examined hepatic GSDMD levels in the liver. Our results showed that both pro-GSDMD and cleaved-GSDMD levels were increased in HFD group and HFD + Al group, and HFD plus ethanol binge facilitated more expression and maturation of GSDMD than HFD alone (Figure 6B).

**FIGURE 7:**
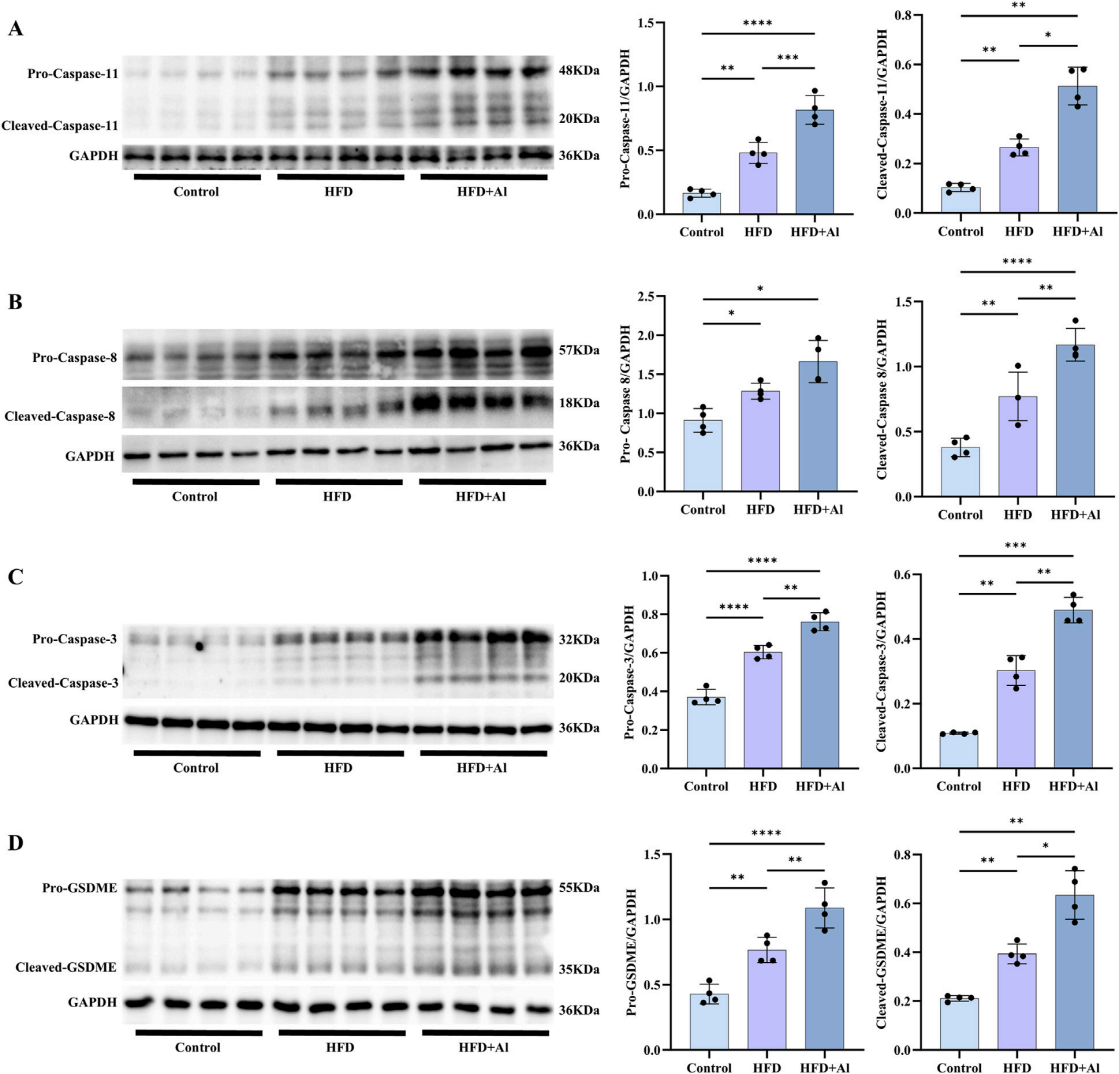
Short-term HFD feeding plus acute ethanol binge increased both the expression and activation of Caspase-11, Caspase-8, Caspase-3 and GSDME in the liver of mice. Western blotting and quantification of pro-Caspase-11/GAPDH and cleaved-Caspase-11/GAPDH **(A)**, pro-Caspase-8/GAPDH and cleaved-Caspase-8/GAPDH **(B)**, pro-Caspase-3/GAPDH and cleaved-Caspase-3/GAPDH **(C)**, and pro-GSDME/GAPDH and cleaved-GSDME/GAPDH **(D)**. All data were expressed as mean ± SD, n = 4 in each group, ^*^p < 0.05, ^**^p < 0.01, ^***^p < 0.001, ^****^p < 0.0001.

In addition to the Caspase-1 to GSDMD canonical pyroptosis signaling, Caspase-8 and Caspase-11 can also induce the non-canonical pyroptosis signaling by cleaving GSDMD, and Caspase-3 can induce the non-canonical pyroptosis signaling by cleaving gasdermin E (GSDME) (Shi et al., 2015; Wang Y. et al., 2017). Therefore, we further examined the non-canonical pyroptosis signals. Our results showed that both HFD and HFD plus ethanol binge can increased the hepatic levels of pro-Caspase-11 and cleaved-Caspase-11 (Figure 7A), pro-Caspase-8 and cleaved-Caspase-8 (Figure 7B), pro-Caspase-3 and cleaved-Caspase-3 (Figure 7C), and pro-GSDME and cleaved-GSDME (Figure 7D). Except for the levels of pro-Caspase-8, which has no statistical significance, other non-canonical pyroptosis signals in HFD + Al group were higher than these in HFD group (Figure 7). Therefore, HFD plus ethanol binge synergistically induced hepatic pyroptosis by excessively activating pyroptosis signals.

## 4 Discussion

The increased adoption of a Western diet, sedentary habits, and alcohol consumption, lead to a rapid increase in the global prevalence of MASLD and ALD (Babuta et al., 2024b). It has been reported that long-term or short-term HFD feeding plus acute ethanol binge synergistically induced liver injury and hepatic steatosis in mice (Chang et al., 2015; Wang W. et al., 2017). In this study, we confirmed that mice received a single dose of ethanol on the 3rd day of HFD feeding enhanced liver injury when compared with the mice only feeding with a HFD for 3 days. Similar to our study, a recent study also showed that short-term feeding of a metabolic-dysfunction-associated steatohepatitis (MASH) diet (high fat (33 gm%), high cholesterol (10 gm%), and high sucrose (208.4 gm%)) plus daily 5 g/kg alcohol gavage for 3 days can induce liver injury in mice (Babuta et al., 2024c). Male mice feeding the same MASH diet combined with receiving 10% alcohol in drinking water *ad libitum* and 5 g/kg alcohol gavage weekly for 3 months displayed the key features of severe alcohol-associated hepatitis (Babuta et al., 2024b). Only weekly alcohol binges (5 g/kg) can also exacerbate liver injury in mice model of MASH received the same MASH diet for 3 months (Babuta et al., 2024a). Moreover, Western diet and alcohol consumption coexist as synergistic insults in a substantial proportion of liver disease patient population (Babuta et al., 2024b). Therefore, HFD combined with habitual alcohol consumption can synergistically cause liver damage.

The synergistic effects of short-term HFD feeding plus acute ethanol binge-induced acute liver injury were involved in hepatic oxidative stress, as that hepatic 3-NT and MDA, which are essential biomarkers of oxidative injury, were enhanced in HFD + Al group when compared with HFD group. In contrast, the antioxidant reduced GSH was downregulated by HFD, or HFD plus acute ethanol binge, and the GSH levels was slightly low in HFD + Al group than HFD group with no statistical significance. These data indicated that short-term HFD feeding plus acute ethanol binge enhanced oxidative stress but decreased the antioxidant capacity in the liver. MPO, which is a member of heme peroxidase family in neutrophils, can generate powerful oxidizing species including hypochlorous acid (HOCl) (Hawkins and Davies, 2021), which also reflects the degree of oxidative stress and inflammation in the organ or tissue. We found that hepatic MPO levels increased in HFD group and HFD + Al group, and there was statistical significance between HFD + Al group and Control group. This was consistent with a previous report that MPO^+^ neutrophils were diffused in the parenchymal regions at 9 h post ethanol gavage in 3d-HFD-fed mice (Chang et al., 2015). Therefore, short-term HFD feeding plus acute ethanol binge may synergistically contribute to liver oxidative stress and inflammation.

The excessive ROS can contribute to the activation of innate immune signals, such as MAPK and NF-κB (Ryan et al., 2004; Tsung et al., 2007; Zhang et al., 2017). Our results showed that short-term HFD feeding plus acute ethanol binge can increase the phosphorylation of ERK, p38, JNK and NF-κB-p65 in the liver. The phosphorylation of MAPK and NF-κB may initiate the production of inflammatory cytokines (Zhang et al., 2017). Our results demonstrated that 3 days HFD feeding plus acute ethanol binge significantly enhanced the protein levels of TNF-α, IL-1β, and IL-18 in the liver of mice. Similar to this, the increased hepatic free fatty acids (FFAs) may contribute to the elevation of *Cxcl1* mRNA in hepatocytes (and to a lesser extent in hepatic stellate cells and sinusoidal endothelial cells) via activating ERK1/2, JNK or NF-κB in mice with 3-day HFD-plus-ethanol binge feeding (Chang et al., 2015). Therefore, the inflammatory cytokines TNF-α, IL-1β, and IL-18 may combine with CXCL1 to synergistically induce liver injury and steatosis.

The inactive precursors of IL-1β and IL-18 should undergo cleavage and activation by the cleaved-Caspase-1, resulting in the formation of mature cleaved forms—cleaved-IL-1β and cleaved-IL-18, which subsequently release from cells to trigger an inflammatory response (Zhang et al., 2017). Our data showed that both the full length of IL-1β and IL-18 (pro-IL-1β and pro-IL-18) and the cleaved IL-1β and IL-18 were increased by HFD plus acute ethanol binge. Szabo et al. found that 3 days combined insult of a MASH-inducing diet and alcohol binges activated hepatic NLRP3 inflammasome, as indicated by a significant increase in the levels of cleaved-Caspase-1 and cleaved-IL-Iβ in the liver (Babuta et al., 2024c). Here, we showed that a short-term HFD feeding with acute ethanol binge markedly elevated the protein levels of both precursor and mature forms of Caspase-1 in the liver of mice. In addition, the activated Caspase-1 may also cleave GSDMD, which is the common effector for cytokine secretion and the typical pyroptosis trigger that follows the activation of inflammasomes (Du et al., 2024). Both GSDMD and GSDMD-N were upregulated in the liver tissues of human MASLD/MASH, and GSDMD plays a key role in the pathogenesis of steatohepatitis, by controlling cytokine secretion, NF-κB activation, and lipogenesis (Xu et al., 2018). Here, we showed that both short-term HFD feeding and short-term HFD feeding plus acute ethanol binge can increase the precursor and maturation of GSDMD in the liver of mice. However, a recent study showed that 3 days MASH diet feeding (which was composed of high fat (33 gm%), high cholesterol (10 gm%), and high sucrose (208.4 gm%)) plus daily acute alcohol binges for 3 days fail to activate GSDMD (Babuta et al., 2024c). This difference may be related to the composition of diet, frequency of alcohol consumption, and even the living environment of mice.

It is well-known that Caspase-1 is activated after various typical inflammasome recognizing ligands, moreover, human Caspase-4 and the mouse homologue Caspase-11 and human Caspase-5 can directly recognize bacterial lipopolysaccharide (LPS), both of which trigger pyroptosis via GSDMD (Shi et al., 2015; Liu et al., 2016). Caspase-11-GSDMD pathway in the liver was activated in a hybrid feeding mouse model of alcoholic hepatitis and patients (Khanova et al., 2018; Wang et al., 2018). Caspase-11 promotes 12 weeks HFD feeding induced NAFLD in mice by increasing glycolysis, oxidative phosphorylation, and pyroptosis in macrophages (Drummer et al., 2023). Our results indicated that both the full-length and cleaved forms of Caspase-11 was enhanced by short-term HFD feeding and short-term HFD feeding plus acute ethanol binge. Caspase-8 activation during TAK1 inhibition results in cleavage of both GSDMD and GSDME (Sarhan et al., 2018). Moreover, GSDME can also be cleaved by Caspase-3 in its linker, generating a GSDME-N fragment that perforates membranes and thereby induces pyroptosis (Wang Y. et al., 2017). Both the precursors and mature forms of Caspase-8, Caspase-3 and GSDME were increased by short-term HFD feeding or short-term HFD feeding plus acute ethanol binge. Moreover, short-term HFD feeding plus acute ethanol binge enhanced more hepatic cleaved-Caspase-8, pro- and cleaved-Caspase-3 and pro- and cleaved-GSDME than only short-term HFD feeding in mice. Thus, both the canonical and non-canonical pyroptosis signaling may be an important mechanism for acute liver injury induced by short-term HFD feeding plus acute alcohol binge.

Our study indicated that short-term HFD feeding plus acute ethanol binge induced acute liver injury in mice through increasing oxidative stress, inflammation, and the canonical and non-canonical pyroptosis signaling. In addition to the well-known of oxidative stress and inflammation, pyroptosis may act as a novel therapeutic target for treating liver damage induced by high calorie diet with excessive alcohol consumption in human beings. ROS can activate MAPK and NF-κB, thereby inducing inflammatory cytokines expression, and the maturation of inflammatory cytokines, such IL-1β and IL-18, requires Caspase-1 (Averill-Bates, 2024; Yang et al., 2024). Classically, Caspase-1 can also induce pyroptosis by cleavage of GSDMD (Shi et al., 2015). GSDMD is a member of gasdermins (GSDMs) family, which consist of GSDMA, GSDMB, GSDMC, GSDMD, GSDME and DFNB 59 (also known as pejvakin (PJVK)) in humans (Zheng et al., 2020). Until now, it has been shown that these GSDMs, except DFNB 59, are inducers of pyroptosis (Wang Y. et al., 2017; Hou et al., 2020; Zheng et al., 2020; Zhou et al., 2020; Deng et al., 2022; Privitera et al., 2023; Zhou et al., 2024). However, it is unclear the roles of GSDMA, GSDMB, and GSDMC in acute liver injury induced by short-term HFD feeding plus acute alcohol binge. Moreover, it is currently uncertain which of these three mechanisms, including oxidative stress, inflammation, and pyroptosis, is more important. It should be noted that MAPK and NF-κB can also be activated independently of ROS, such as by the increased LPS (Carpino et al., 2020). Therefore, it is still uncertain which of these three mechanisms is the main one? More likely, they have both upstream-downstream relationships and can independently play a role in liver damage.

## Data Availability

The raw data supporting the conclusions of this article will be made available by the authors, without undue reservation.
